# Altered Behavior and Neuronal Activity with Paternal *Snord116* Deletion

**DOI:** 10.3390/genes16080863

**Published:** 2025-07-24

**Authors:** Daniel S. Scott, Violeta Zaric, Carol A. Tamminga, Ryan K. Butler

**Affiliations:** 1 Department of Psychiatry, UT Southwestern Medical Center, Dallas, TX 75390, USA; daniel.scott2@utsouthwestern.edu (D.S.S.); carol.tamminga@utsouthwestern.edu (C.A.T.); 2 Department of Pediatrics, UT Southwestern Medical Center, Dallas, TX 75390, USA; violeta.zaric@utsouthwestern.edu; 3 O’Donnell Brain Institute, UT Southwestern Medical Center, Dallas, TX 75390, USA

**Keywords:** Snord116, Prader–Willi syndrome, memory, psychosis, anxiety

## Abstract

Background/Objectives: Prader–Willi Syndrome (PWS) is a neurodevelopmental disease associated with multiple behavioral features, including a prevalence for psychosis. The genetic causes of PWS are well characterized and involve the silencing or deletion of the paternal copy of a region of chromosome 15q11–13. One gene within this region, *Snord116*, a non-coding RNA, has been determined to have a determinant role in the manifestation of PWS. However, it remains unclear as to how the deletion of this allele can affect activity in the brain and influence psychosis-like behaviors. Methods: In this study, we assessed the effects of the microdeletion of the paternal copy of *Snord116* on regional neural activity in psychosis-associated brain regions and psychosis-like behaviors in mice. Results: The results suggest that *Snord116* deletion causes increased c-Fos expression in the hippocampus and anterior cingulate cortex. *Snord116* deletion also results in behavioral phenotypes consistent with psychosis, most notably in stressful paradigms, with deficits in sensorimotor gating and augmented contextual as well as cued fear conditioning. Conclusions: These results implicate the targets of *Snord116* in the presentation of a psychosis-like state with regional specificity.

## 1. Introduction

Prader–Willi Syndrome (PWS, OMIM176270) is a neurodevelopmental disorder characterized by early childhood obesity, hyperphagia, hypotonia with poor suck and poor weight gain in infancy, mild to moderate intellectual disability, hypogonadism, growth hormone insufficiency, and psychiatric disturbances. Schizophrenia and psychotic illness are of much higher prevalence in the PWS population compared to the general population with rates dependent on the genetic subtype of PWS. In the general population, studies on the genetic underpinnings of schizophrenia/psychosis suggest a multitude of genetic factors along with environmental confounders. Studies on PWS offer the opportunity to decipher the role(s) of specific genetic loci and how they emerge as the cellular, neurophysiological, and behavioral features of psychosis. Prior studies have demonstrated that rodents with the deletion of several PWS genes display behaviors with face validity for psychotic illness [1,2]. It is unknown if these behaviors are also present when the genetic deletion is specific to the small nucleolar RNA C/D box 116 (Snord116)—a tandemly repeated non-coding RNA that modifies other small nuclear RNAs. Furthermore, it is unknown if brain regional phenotypes associated with psychosis, such as hyperactivity of the anterior cingulate cortex (ACC) and hippocampus, are present in paternal Snord116 deletion mice.

PWS is caused by the loss of function of imprinted genes normally expressed on the paternal allele located in the chromosomal region 15q11–q13. This can occur either through the deletion of the chromosomal region from the paternal allele, maternal uniparental disomy, or an imprinting center defect, where epigenetic defects result in the silencing of the paternal allele. Patients with a small deletion of 108 kb encompassing the *SNORD116* cluster appear to have most of the PWS-related clinical phenotypes, demonstrating the importance of *SNORD116* in PWS pathogenesis [3,4,5,6]. Furthermore, females with SNP and haplotype variations in *SNORD116* demonstrate increased paranoia associated with schizotypal traits in a non-clinical population [7]. The genetic targets of *SNORD116* are elusive, as are the brain region-specific consequences. However, a recent study uncovered 42 genes that appear to be regulated by *SNORD116* [8]. Many of these gene targets have been shown to be associated with psychosis-like features in human populations and animal models. Features of psychosis include hyperactivity of cortical regions such as the anterior cingulate cortex (ACC) and hippocampus [9,10]. The anterior cingulate cortex (ACC) is an important brain region in facilitating general motor and affective behavior [11], the retrieval of remote memory [12], and the integration of reward as well as memory information to influence behavior [13], and dysfunction of the ACC is implicated in schizophrenic and psychotic disorders [9,14]. Moreover, antipsychotic treatment can reduce ACC hyperactivity at rest in people with schizophrenia, and this effect is associated with improvement in the severity of psychosis [15]. Similarly, the hippocampus is a critical brain region mediating multiple processes, including affective behaviors, learning, episodic memory, social function, and spatial navigation, and shows volumetric changes as well as hyperactivity in psychotic disorders. Here, we seek to assess basal ACC and hippocampal activity, as measured by c-Fos expression, in *Snord116* deletion mice. 

Prior studies have demonstrated that PWS mouse models display similar behavior phenotypes to psychosis mouse models [2,16], though it is unclear how these effects are associated with alterations in regional neural activity. Our lab has shown hippocampal hyperactivity in psychosis patients and rodent mouse models of psychosis [10,17,18]. In this study, we sought to assess basal activity in the ACC and hippocampal subfields in mice with the paternal deletion of *Snord116* and assess psychosis-like behaviors in several domains, including motor and affective behaviors, learning and memory behaviors, and sensorimotor gating. We hypothesize that *Snord116* deletion will recapitulate the behaviors of relevant psychosis mouse models, and that brain regional hyperactivity will be present in these *Snord116* deletion mice. 

## 2. Materials and Methods

Animals: All animal studies were conducted in accordance with IACUC and under UT Southwestern Medical Center protocol 102619. All studies utilized C57BL/6N Snord116^p−/m+^ mice (B6.Cg-Snord116tm1.1Uta/J, stock 008149) developed by the Francke lab, which were obtained from the Jackson Laboratory and have since been backcrossed onto a C57Bl/6N background over > 10 generations. Snord116^p−/m+^ study mice (which carry a paternally inherited chromosome 7 lacking the Snord116 gene cluster) and WT littermates were generated by crossing male C57Bl/6N Snord116^p−/m+^ mice with female C57BL/6N mice. After weaning at P28, animals were group-housed with their same-sex littermates. Housing conditions included a 12 h light–dark cycle and ad libitum water access. Both male and female mice were used in all experiments. Animals underwent behavioral testing at approximately 4 months of age, and were then sacrificed for tissue analysis at approximately 6 months of age. Mice were observed for behaviors such as feeding and body weight changes, previously determined to be deficient in this model, to ensure the induction of the disease. We looked for significant signs of distress and a reduction in normal activities. If any mouse showed sudden weight loss and reacted poorly (lethargic, non-responsive to touch, and weight loss > 20%), we euthanized the animal before it experienced more than 20 percent weight loss.

Genotyping: Genotyping was performed using genomic DNA extracted from tail snips and three primers to detect the *Snord116*^p−/m+^ and WT *Snord116* alleles: Snord116-M634 (5′-TGGATCTCTCCTTGCTTGTTTTCTC-3′), Snord116-M635 (5′-AATCCCCAACCTACTTCAAACAGTC-3′), and Snord116-M636 (5′-TTTACGGTACATGACAGCACTCAAG-3′). The PCR protocol generated a WT band of 435 bp and a mutant band of 337 bp.

Immunohistochemistry: Analysis of c-Fos+ cell density was used as a proxy of regional activity. Mice were sacrificed with CO_2_, then transcardially perfused with physiological saline, followed by 4% paraformaldehyde. Brains were passed through a sucrose gradient, then coronally sliced at 50 μm. Every sixth section was quenched free of endogenous peroxidases in 0.3% H_2_O_2_ and blocked in a buffer containing 2% normal donkey serum, prior to a 72 h incubation in rabbit anti-c-Fos polyclonal antibody (1:8000; Synaptic Systems 226-008, Goettingen, Germany). The primary antibody was detected by sequential incubation with biotinylated donkey anti-rabbit IgG (1:1000, Jackson ImmunoResearch, West Grove, PA, USA) and avidin–biotin complex (Vector Laboratories, Burlingame, CA, USA). The immunoperoxidase signal was detected with incubation with diaminobenzidine chromogen with nickel sufate enhancement. Images were captured from mounted sections using a light microscope, and c-Fos+ cell nuclei were counted using ImageJ (Version 1.54g). The total number of c-Fos+ cell nuclei divided by the area of the region was used to determine c-Fos+ cell density. Due to differences in staining efficiency between antibody lots and experimental runs, data were normalized to the average of the control animals of each sex in each cohort. To assess c-Fos+ cell density in the ACC, sections spanning the anterior–posterior extent of the ACC, from approximately 2.34 mm anterior of bregma to 0.38 mm anterior of bregma, were analyzed. For the hippocampus, we assessed the extent from approximately 1.06 mm to 3.52 mm posterior to bregma. The quantification of c-Fos+ cell density was performed by an experimenter blind to the IDs of the animals within each cohort.

Behavioral Battery: Mice were run through a battery to assess both general motor and affective behaviors, as well as behaviors associated with a psychosis-like phenotype and/or hippocampal dysfunction. Behavioral tests were separated by at least 48 h, and were performed over a period of 2 weeks. The order of behaviors was kept constant, with the least stressful paradigms being performed first, and the most stressful last. The order used was: (1) open field; (2) social memory; (3) spontaneous alternation; (4) prepulse inhibition; and (5) contextual as well as cued fear conditioning. Behavior testing was performed by an experimenter blind to the genotypes of the animals within each cohort.

Open Field: To assess general motor function and anxiety, animals were placed into a large open field, measuring 45 cm × 45 cm, with 30 cm high walls. Animals were allowed to freely explore the open field in dim light (50–55 lumens), and their position was recorded with an overhead camera. Using Ethovision software, locomotor activity and thigmotaxis were assessed.

Social Memory: Mice were habituated to an empty cage for 15 min, after which a sex-matched 4-week-old C57BL/6J mouse was placed in the cage for 2 min; the time the resident mouse spent in contact/sniffing, following, nosing/grooming, or pawing/general inspection was quantified. The procedure was repeated 24 h later, introducing the same juvenile mouse to the same resident. The difference in time between the first and second tests is used as a measure of social memory.

Spontaneous Alternation: For this task, we used a Y-maze, with arms offset by 120°. Arms were 30 cm long and 6 cm wide, with walls 15 cm high. At the beginning of each trial, mice were confined in the home arm for 5 s, at which point a door was opened to allow the mouse to explore the apparatus for 2 min. Upon entering either goal arm, a door was lowered to confine the animal within that arm for 30 s, ending the trial. The animal was then returned to the starting position to begin the next trial. If the animal did not enter either goal arm during the 2 min trial, the animal was returned to the home arm and confined there for 30 s, and no choice was recorded. Successful alternation is defined as the entering of the opposite arm from the last trial in which a choice was made. Each session consists of eleven trials, providing ten opportunities for alternation.

Prepulse Inhibition (PPI): Mice were placed inside a tube in a sound-attenuated chamber (SR-Lab, Sand Diego Instruments, San Diego, CA, USA) with a background white noise played at 70 dB. Mice were allowed to habituate to the chamber for five minutes, then were exposed to a 120 dB 40 msec pulse of white noise as a startle, or the startle preceded by a 20 msec prepulse at 72, 74, 78, or 82 dB in a pseudorandom order, with an average intertrial interval of 15 s (range 7–23 s). The degree to which the response amplitude was inhibited by the presentation of the prepulse was calculated as [1−(prepulse trials/startle-only trials)] * 100, representing % inhibition.

Fear Conditioning: Mice were placed in a novel conditioning apparatus (Med Associates, St. Albans, VT, USA) consisting of a clear, Plexiglas chamber with a metal bar floor. During the six-minute training session, mice were exposed three times to an 80 dB, 30 s auditory cue (CS), co-terminating with a 0.5 mA foot shock.

Contextual Fear Conditioning: Twenty-four hours after training, mice were returned to the same chamber, and the percentage of time the animal was motionless (freezing) over the five minute trial was recorded.

Cued Fear Conditioning: Twenty-four hours following the contextual fear conditioning trial, mice were placed in a novel environment, consisting of black side walls, a solid plastic floor, and scented with vanilla for six minutes. After three minutes, the CS was played for the remainder of the session. The percentage of time spent freezing either before (pre-CS) or during (CS) the cue presentation was recorded.

Statistical AnalysiFor all experiments, N = 38 mice in the WT condition (19 male and 19 female), and N = 22 in the *Snord116*^p−/m+^ condition (12 male and 10 female), with the exception of c-Fos analysis in ACC, where one WT animal was excluded from the analysis due to damage to the ACC upon the removal of the brain from the skull. This number was determined entirely by the makeup of the litters allocated for the designed experiments. A Gaussian distribution of data was determined by using the Kolmogorov–Smirnov test. In cases where the data did not fit a normal distribution, a minimal number of outliers were removed from the dataset, altering the final *n* for some experiments. The specific N for all experiments is described in Table S1. In no case did the removal of outliers alter the significance of the parametric statistical tests used for analysis. In all statistical analyses, significance was defined at *p* < 0.05. For all analyses where statistical significance was determined, the statistical results are described in the Results section of this manuscript.

For immunohistochemistry experiments, data were analyzed with multi-factor ANOVA, with factors of sex, genotype, and, in the case of the hippocampus, subfield. When appropriate, post hoc analyses were performed using Fisher’s LSD test. In cases where no main effect or interaction with sex was determined, sexes were pooled and presented together.

For behavioral experiments, all data were analyzed using multi-factor ANOVA, with factors of sex and genotype, and, when appropriate, a repeated measure of prepulse intensity (PPI), or test phase (cued fear conditioning). When appropriate, post hoc analyses were performed using Fisher’s LSD test. All statistical analyses were performed using Prism software (Version 10.5.0.774; GraphPad Software, Boston, MA, USA). In cases where no main effect or interaction with sex was determined, sexes were pooled and presented together. Descriptive statistics of all data are presented in Table S1.

## 3. Results

### 3.1. Regional Activity

**Figure 1:**
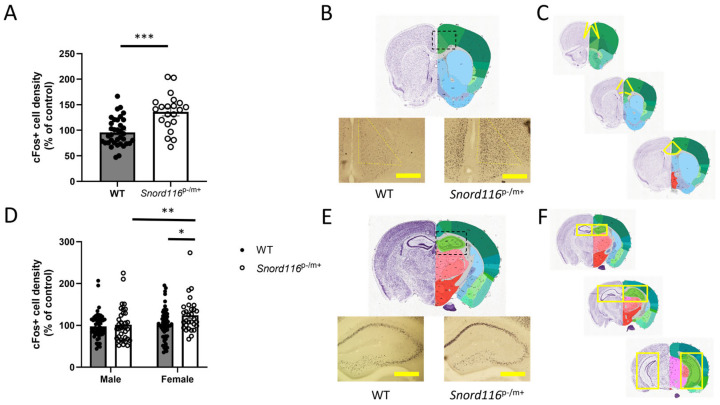
Quantification of c-Fos+ cell density as a proxy of neuronal activity. (**A**–**C**) c-Fos+ cell density in the anterior cingulate cortex (ACC). (**A**) Quantification of c-Fos+ cell density in WT and *Snord116*^p-/m+^ mice, including both male and female mice. *** represents *p* < 0.001. (**B**) Representative images of c-Fos staining in WT (**left**) and *Snord116*^p−/m+^ (**right**) mice. (**C**) Images demonstrating the anterior (top) and posterior (bottom) extent of analysis within the ACC. Yellow areas indicate the region of interest. (**D**–**F**) c-Fos+ cell density in the hippocampal subfields of dentate gyrus (DGs), cornu ammonis 3 (CA3), and cornu ammonis 1 (CA1). (**D**) Quantification of c-Fos+ cell density in WT and *Snord116*^p-/m+^ mice, separated by sex. ** represents *p* < 0.01. (**E**) Representative images of c-Fos staining in WT (**left**) and *Snord116*^p-/m+^ (**right**) mice. (**F**) Images demonstrating the anterior (top) and posterior (bottom) extent of analysis of the hippocampus. Yellow areas indicate the region of interest. * represents a main effect of genotype, *p* < 0.05. A main effect of sex was also detected (*p* < 0.05). Scale bar: 500 μm. Dotted boxes refer to the general area where c-Fos was measured. Reference images from the Allen Mouse Brain Atlas, mouse.brain-map.org and atlas.brain-map.org (accessed 1 May 2025).

To assess basal levels of neural function within specified brain regions, we quantified the density of cells expressing c-Fos, an immediate early gene that can be used as a proxy of recent neuronal activity. We assessed c-Fos+ cell density in two distinct regions associated with PWS or psychosis: the anterior cingulate cortex (ACC) and the hippocampus, separately quantifying c-Fos+ cell density in the different hippocampal subfields. To quantify these data, cell density was normalized to the count from all WT mice within each staining cohort, and these normalized values were contrasted between male and female mice to determine if there was an effect of sex on activity within these regions. In analyses of ACC, data from one WT male and one *Snord116*^p−/m+^ female were removed as outliers. In the ACC, our statistical analyses revealed no effect of sex (F(1,55) = 0.3061, *p* = 0.5824), so data from male and female mice were pooled. We did detect a main effect of genotype (F(1,55) = 12.81, *p* = 0.0007) indicating that *Snord116*^p−/m+^ mice show a significant increase in c-Fos+ cell density relative to WT mice (Figure 1A,B).

In analyses of the hippocampus, data from CA1 of one *Snord116*^p−/m+^ male was excluded as an outlier. In the hippocampus, we did detect a main effect of sex F(1,165) = 4.161, *p* = 0.043), so male and female WT and *Snord116*^p−/m+^ mice were assessed separately. We further found a main effect of genotype (F(1,165) = 5.782, *p* = 0.0173), suggesting an increase in hippocampal activity in *Snord116*^p−/m+^ mice relative to the WT (Figure 1C,D). Moreover, we saw no effect of subfield (F(2,165) = 0.8672, *p* = 0.422) or subfield x genotype interaction (F(2,165) = 0.7719 *p* = 0.4638), indicating that this effect is consistent throughout the hippocampal subfields, so data from the subfields were combined for presentation. As there was no sex x genotype interaction (F(1,165) = 1.216, *p* = 0.2717), these results suggest that although female mice may show greater hippocampal activity in general compared with male mice, *Snord116* deletion similarly affects hippocampal function in both sexes, causing hippocampal hyperactivity.

### 3.2. Behavioral Analyses 

Because alterations of function in the hippocampus and ACC can contribute to a variety of behavioral outcomes, including increased anxiety, effects on learning and memory, and psychosis-like behaviors, we sought to assess the behavioral consequences of *Snord116* deletion with a particular focus on these specific domains. 

#### 3.2.1. Motor and Affective Behaviors 

**Figure 2:**
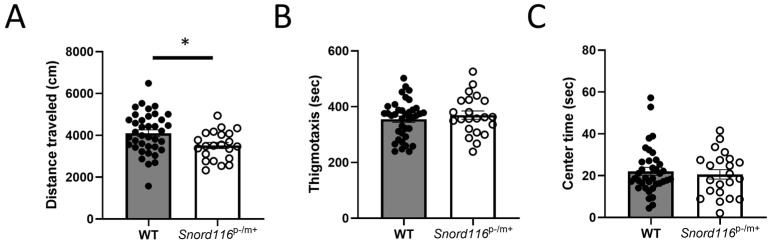
Behavioral analyses: motor and affective behaviors. (**A**) Locomotor activity was assessed by measuring the distance traveled in an open field in both male and female mice. * represents *p* < 0.05, indicating decreased general motor activity in *Snord116*^p−/m+^ mice relative to the WT. Anxiety was assessed by analyzing (**B**) the time spent in the periphery (thigmotaxis) or (**C**) in the center of the open field in both male and female mice.

We first assessed the behavior of WT and *Snord116*^p−/m+^ mice in an open field, assessing the general motor function of both, to determine whether any phenotype observed can be explained by changes in overall activity, as well as thigmotaxis and time spent in the center of the arena, two measures that can assess general anxiety. In analyses of time spent in the center of the arena, data from one WT male was excluded as an outlier. We saw no main effect or interaction with sex in any of these outcomes, so data from male and female mice were pooled. Interestingly, *Snord116*^p−/m+^ mice showed decreased locomotor activity relative to WT mice (Figure 2A), while there was no effect of genotype on either measure of anxiety, with *Snord116*^p−/m+^ mice showing equivalent time in the periphery (Figure 2B) or center of the arena (Figure 2C).

#### 3.2.2. Learning and Memory

We next assessed the memory functions in WT and *Snord116*^p−/m+^ mice, subjecting the animals to tasks that test associative memory (contextual as well as cued fear conditioning), spatial working memory (spontaneous alternation), and social memory. Again, we detected no main effect or interaction with sex in any of these paradigms, so data from male and female mice are pooled for presentation.

**Figure 3:**
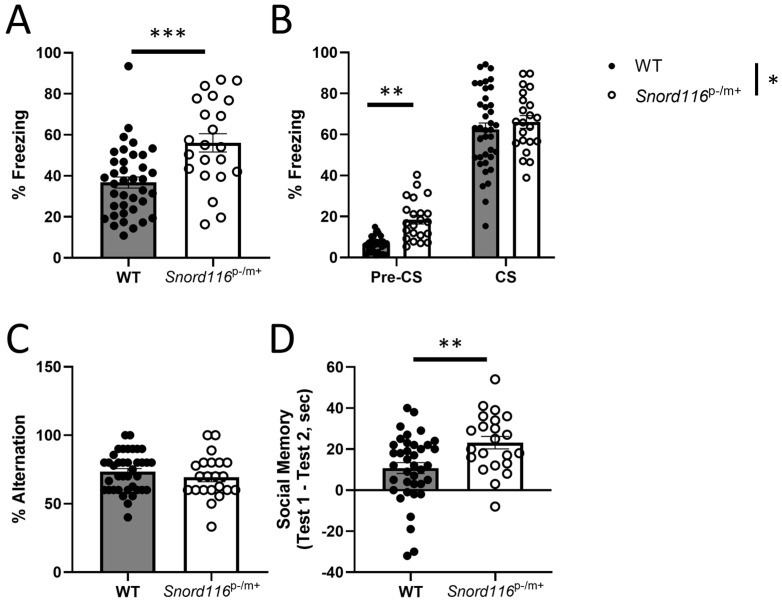
Behavioral analyses: learning and memory. (**A**,**B**) Associative memory was assessed with contextual and cued fear conditioning in both male and female mice. (**A**) Contextual fear conditioning in WT and *Snord116*^p−/m+^ male and female mice. *** represents *p* < 0.001, indicating increased freezing in a shock-associated context in *Snord116*^p−/m+^ mice. (**B**) Cued fear conditioning in a novel context both prior to (pre-CS) and during (CS) presentation of the CS. * represents a main effect of genotype, indicating greater total freezing in *Snord116*^p−/m+^ mice relative to controls. ** represents *p* < 0.01, indicating increased freezing in *Snord116*^p−/m+^ mice prior to CS presentation relative to controls. (**C**) Spatial working memory was assessed in both male and female mice by analyzing spontaneous alternation in a Y-maze. (**D**) Social memory was measured in both male and female mice by analyzing the difference in time spent interacting with a sex-matched juvenile mouse in two tests 24 h apart. ** represents *p* < 0.01, indicating a greater degree of social memory in *Snord116*^p−/m+^ mice relative to the WT.

In analyses of contextual fear conditioning, data from one WT male was removed as an outlier, and for pre-CS freezing in the cued fear conditioning test, four WT males and one WT female were excluded. Results from the contextual fear conditioning tests demonstrate a main effect of genotype (F(1,55) = 17.77, *p* < 0.0001), indicating increased freezing of the *Snord116*^p−/m+^ mice relative to the control (Figure 3A). Moreover, we determined a main effect of genotype in the cued fear conditioning (F(1,58) = 6.624, *p* = 0.0126), but no test phase x genotype interaction (F(1,53) = 2.655, *p* = 0.1092). A priori comparisons revealed that this effect is due to *Snord116*^p−/m+^ mice freezing more than WT mice prior to CS presentation (Figure 3B).

In contrast to this effect, we saw no effect of *Snord116* deletion on spatial working memory in the spontaneous alternation task (F(1,56) = 1.038, *p* = 0.3127) (Figure 3C). To assess social memory, we measure the amount of time the experimental mouse spent interacting with a sex-matched juvenile for two minutes on two occasions, 24 h apart. The magnitude of the difference in the interaction time between the test days is indicative of the degree of social memory.

Consistent with the heightened performance seen in the fear conditioning tests, we determined that *Snord116*^p−/m+^ mice show a greater degree of social memory compared to their WT counterparts (F(1,56) = 8.627, *p* = 0.0048) (Figure 3D). These results suggest that *Snord116* deletion does not affect the temporary storage of spatial memory as assessed with the spontaneous alternation task, while behavior in tasks that involve a longer period between learning and recall is augmented, perhaps implicating memory consolidation.

#### 3.2.3. Prepulse Inhibition (PPI)

**Figure 4:**
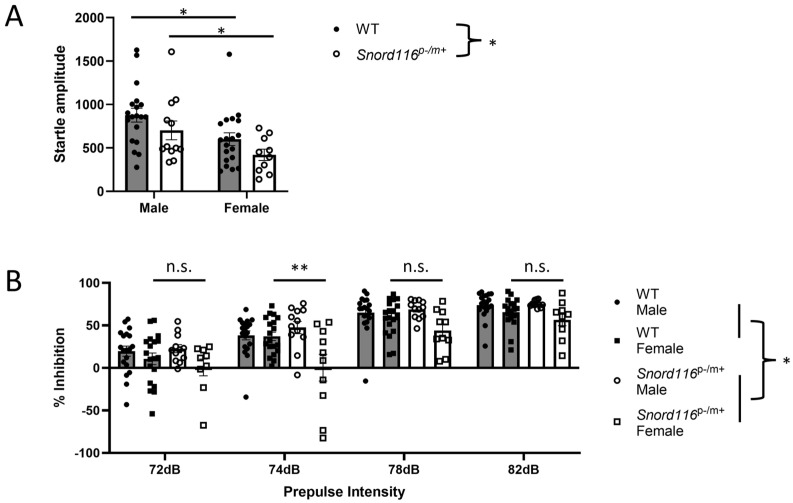
Behavioral analyses: prepulse inhibition of an acoustic startle response. (**A**) Startle amplitude to a 120 dB acoustic stimulus. * represents *p* < 0.05. (**B**) Sensorimotor gating was assessed by analyzing prepulse inhibition (PPI) separately in male and female mice. We detected a main effect of genotype (*p* < 0.05), suggesting impaired PPI in *Snord116*^p−/m+^ mice relative to the WT (*p* < 0.05), indicated on the right by *. A main effect of sex and a sex x genotype interaction were also determined (*p* < 0.05), and post hoc analysis determined a significant impairment in PPI in female *Snord116*^p−/m+^ mice relative to the WT at a 74 dB prepulse intensity, indicated by ** (*p* < 0.01). No significant difference was detected between WT and *Snord116*^p−/m+^ mice at any other prepulse intensity (n.s.).

As directly assessing psychosis in an animal model is particularly difficult, due to the subjective nature of psychosis, we assessed the prepulse inhibition of an auditory startle, a measure of sensorimotor gating that is similarly affected in humans with psychosis and animal models of psychosis, either induced with psychotomimetic treatment or generated based on psychosis-related genetic or environmental factors. Auditory startle data from two *Snord116*^p−/m+^ male mice, and PPI data from one *Snord116*^p−/m+^ female in trials with a 72 dB prepulse and one *Snord116*^p−/m+^ male in trials with an 82 dB prepulse, were removed as outliers. When assessing the startle response in the absence of a prepulse, we detected a significant effect of sex (F(1,56) = 10.12, *p* = 0.0024) and genotype (F(1,56) = 4.112, *p* = 0.0473), but not sex x genotype interaction (F(1,56) = 0.0001, *p* = 0.9748). Post hoc comparisons indicate that male mice show a greater startle response than female mice regardless of genotype, while no difference was detected between WT and *Snord116*^p−/m+^ mice of the same sex (Figure 4A). When assessing prepulse inhibition, we found a main effect of genotype (F(1,222) = 4.401, *p* = 0.037), sex (F(1,222) = 22.19, *p* < 0.0001), as well as a sex x genotype interaction (F(1,222) = 12.07, *p* = 0.0006). Post hoc analyses demonstrate that female *Snord116*^p−/m+^ mice show a significant impairment in PPI relative to female WT mice at a prepulse 4 dB above the background noise, while male *Snord116*^p−/m+^ mice were indistinguishable from their WT counterparts regardless of prepulse intensity (Figure 4B). These results suggest that female animals, already demonstrating impairments in sensorimotor gating, consistent with previous results, are particularly susceptible to further disruptions in the ability to incorporate sensory information to regulate motor responses. 

## 4. Discussion

Our results indicate that a Snord116 deletion in mice results in psychosis-like behavior and is sufficient to increase c-Fos expression in the anterior cingulate cortex and hippocampus, suggesting regional neuronal hyperactivity. In the ACC, we measured an increase in c-Fos expression regardless of sex. In the hippocampus, we also measured detected increased c-Fos expression in female mice, regardless of genotype, with a further increase in activity seen with Snord116 deletion. Behavioral alterations observed in Snord116^p−/m+^ mice include decreased activity in an open field, augmented contextual and cued fear conditioning, improved social memory, and impairments in sensorimotor gating. Taken together, these data suggest that Snord116 deletion recapitulates many similar features found in psychosis mouse models and provides additional detail on the genetic underpinnings of psychosis. 

The ACC and the hippocampus share robust bi-directional connectivity, particularly between the ACC and the dorsal CA3/CA1 subfields of the hippocampus [19]. We have shown elsewhere that with this same *Snord116* deletion, we can observe cell-autonomous effects in the ACC, including increased spontaneous excitatory currents and alterations in resting membrane potential and resistance. The hyperexcitable ACC is likely to mediate the increased c-Fos expression in the hippocampus we observed in this study. Further analysis to determine whether *Snord116* deletion similarly affects the cell-autonomous properties of hippocampal granule cells and pyramidal cells is warranted and could suggest the inverse: that increased ACC activity is the result of increased hippocampal excitability. The increased hippocampal c-Fos expression in female mice relative to male mice, regardless of genotype, may reflect the consequence of greater dendritic complexity in CA3/CA1 in female animals relative to males, as described in previous studies [20,21,22,23]. However, it should be noted that in humans with PWS, a number of additional brain regions show volumetric differences from the control population, including the amygdala, thalamus, and brainstem [24]. Analysis of these regions was beyond the scope of the current study, but future analyses will assess the functional outcomes in these regions, as well as other, novel regions, which can be determined through unbiased functional and volumetric analyses in these animals, including structural and functional MRI. 

Our behavioral tests were designed to test three behavioral domains: motor and affective behaviors, learning and memory, and sensorimotor gating. These specific domains were assessed for several reasons: first, PWS patients show aberrant behavior in tasks associated with these domains, so the analysis of these phenotypes would be necessary to provide some degree of face validity to this model of PWS. Second, the proper functioning of the hippocampus and anterior cingulate cortex are necessary for normal behavior in tasks that utilize these features. With changes in the basal function of these regions, it stands to reason that some or all of these domains can be disrupted in *Snord116* deletion mice and may provide a direct link between regional changes in neuronal activity and specific behavioral phenotypes. Finally, it is of particular interest that we attempt to tease out the features of altered behavior that could lead to the neuropsychiatric effects of the disease, specifically psychosis-like behavioral manifestations. Psychosis is a very complex phenomenon, characterized by deficits in all five domains associated with neuropsychiatric conditions (negative and positive valence systems, cognitive systems, social function, arousal/regulatory systems) [25], which include alterations in memory, social function, and sensory gating [26,27]. By using a comprehensive behavioral battery, we can determine the specific features of psychosis affected by *Snord116* deletion, which could contribute to a psychiatric presentation. 

We first assessed activity in an open field to determine whether *Snord116* deletions result in changes in the locomotor response to a novel environment or demonstrate general anxiety. *Snord116* deletion mice showed a decrease in activity, without a difference in the total time spent in either the periphery (thigmotaxis) or center of the arena, which would suggest no difference in general anxiety, but with less total activity. The effect seen on general activity is consistent with what is seen in humans with PWS, who demonstrate hypotonia, muscle weakness, and delayed motor function as newborns [28,29], and this decrease in activity is still present in adult patients [30]. Interestingly, this effect was not reported in another study using the same animal model [31]; however, in that study, mice were assessed at 7 weeks of age, as opposed to 4 months of age in this study, offering one potential explanation for the different outcomes. It has been shown that people with PWS are more likely to show decreased movement with age [32,33]. Alternatively, it is possible that this discrepancy is due to the order of behavioral testing between the two studies. We exposed animals to the open field as the first behavioral paradigm, while this other study ran this behavioral task following several others, including an elevated plus maze and light–dark exploration tasks, which induce a greater level of stress upon the animal, potentially affecting general behavior. The lack of an anxiogenic phenotype is a somewhat surprising finding; in humans with PWS, anxiety is a common behavioral symptom, often triggered by changes in routine [34]. The placement of the mice into a novel open field would be likely to represent such a change in routine and the fact that we did not see decreased time in the center of the arena, or increased thigmotaxis, in *Snord116*^p−/m+^ mice would belie an increase in anxiety. However, due to the broad nature of the open-field test, it is plausible that the decrease in total activity, rather than representing a strictly locomotor effect, may further be the result of increased anxiety, causing increased immobility in response to stress. Further studies assessing locomotor activity in the home cage, or repeated exposures to the open field to reduce the novelty of the arena, are needed to parse out this effect. 

We next assessed measures of learning and memory, as humans with PWS demonstrate learning disabilities. Our analyses sought to assess three distinct memory types: spatial working memory, using the spontaneous alternation task, social memory, and associative memory, assessing the response to both a fear-related cue and context. Spontaneous alternation is premised on the natural tendency of mice to explore a previously unexplored arm of a Y-maze, even without the possibility of reward [35]. We report here that *Snord116* deletion demonstrates no difference from healthy controls in the percentage of trials in which the animal explored the opposite arm of the previous trial. This is consistent with the human presentation of PWS, as it has been reported that people with PWS show little impairment in visual–spatial memory, with stronger performance in spatial tasks when PWS is the result of uniparental disomy [36,37], and do not differ from healthy controls when analyzing the number of errors in a modified radial arm maze task [38]. 

Social memory is tested by quantifying the amount of time a mouse interacts with a juvenile on two consecutive days; the magnitude of the decrease in interaction time in the second test relative to the first is indicative of the strength of social memory. While PWS patients show social deficits, particularly in the recognition of emotional states, humans with PWS caused by maternal uniparental disomy show a habituation in visual-event-related potential responses to repeatedly displayed faces, suggesting the intact recall of a previously encountered social partner, while those with a deletion genotype show no such habituation [39]. Consistent with what is observed with uniparental disomy PWS patients, *Snord116*^p−/m+^ mice show no deficit in social memory, and in fact show a greater magnitude of social memory compared to their wild-type littermates. As social memory is mediated, at least in part, by hippocampal CA2 function [40], the increased hippocampal activity in *Snord116*^p−/m+^ mice may be related to this interesting phenotype. It is important to note that CA2 is a small region between CA3 and CA1, without defining anatomical characteristics to allow for precise analysis, so any effect on c-Fos expression that we may have observed in this subfield would be included with the effects in CA3. 

We also assessed associative memory using the fear conditioning paradigm. This task separately measures the behavioral response to both a context and cue associated with a foot shock. Contextual fear conditioning is dependent on hippocampal, as well as amygdalar, function, while cued fear conditioning remains intact even without the contribution of the hippocampus [41]. As would be predicted from animals with hippocampal hyperactivity, *Snord116*^p−/m+^ mice showed a greater degree of freezing in the contextual fear conditioning test. Increased time spent freezing in the contextual fear conditioning test was also observed in animal preparations displaying hippocampal hyperactivity, which were designed to mimic the pathology seen in the post-mortem analysis of humans with psychosis [15]. Additionally, the acute chemogenetic excitation of the ventral portion of CA3 has also been shown to selectively increase contextual fear conditioning [15], demonstrating the sufficiency of thehyperactivation of the hippocampus in the presentation of this behavioral phenotype. The findings reported here are consistent with that effect and may reflect some of the psychosis-like tendencies seen in PWS [42]. Interestingly, although we see a main effect of genotype in the cued test, the increased freezing was present both prior to and during CS presentation, suggesting the increased freezing in *Snord116*^p−/m+^ mice in this paradigm is not a response to a fear-associated stimulus, but rather a generalized state of anxiety in a novel context. This also differs from a separate study using the same animal model at a younger age [31], which showed decreased freezing to the shock-associated cue, and no difference in contextual freezing. Clinical studies in humans with PWS have shown that older people tend to be slower-moving and lazier, with increased fatigue and decreased movement relative to younger individuals [33,43], which could potentially manifest in the behavioral response to a fear-associated context/cue, leading to the increased freezing detected here. As our findings are consistent with the increased hippocampal activity observed in these animals, it may be of interest to determine how both the behavioral phenotypes and alterations in regional activity change over the life course of this animal model.

Our last behavioral paradigm was prepulse inhibition, a measure of sensorimotor gating that assesses the ability to filter pre-attentive irrelevant sensory stimuli, which is impaired in humans with psychosis [44,45]. As both humans and animals can be similarly tested in this task [46,47], this task provides an assessment of psychosis-like behavior in animal preparations with good face validity. We found that female *Snord116*^p−/m+^ mice show an impairment in PPI relative to WT mice, while male *Snord116*^p−/m+^ mice show no such deficit. This sex difference is unexpected, as the prevalence or severity of psychosis shows no sex difference in the human population, though this type of neuropsychiatric manifestation in PWS is more common with uniparental disomy than with a deletion [42]. However, we did also detect a reduced startle response in *Snord116*^p−/m+^ mice relative to WT mice, and in females relative to males. It is therefore possible that the effects seen in this paradigm are at least partially due to a reduced baseline response in *Snord116*^p−/m+^ mice generally, and female *Snord116*^p−/m+^ mice specifically.

It is important to note that psychosis associated with PWS is atypical, with more mood-related than thought-related features, and increased stress reactivity [42]. As we have seen in this study, a psychosis-like phenotype was most robust in behavioral paradigms that were the most stressful for the animal, including prepulse inhibition, where the animal is kept in a confined space for over 20 min, and fear conditioning, where the animal receives a foot shock. Psychosis is a complex phenomenon with many distinct features encompassing several behavioral domains. Since a number of genes have been shown to have some association with the emergence of a psychotic neuropsychiatric profile in one way or another, it is likely that the unique nature of the psychoses observed in PWS patients is a function of the specific alterations in the constellation of genes targeted by the genotypic alterations in PWS. Recently, a novel network of genetic targets of *Snord116* was postulated [8]. A cross-study comparison (Table S2) suggests that several of these dysregulated genes are also associated with psychosis in humans and/or animal models. What is evident from these findings is that the behavioral phenotypes associated with alterations in the expression or mutations of these genes can be quite diverse. It is plausible that the loss of *Snord116* function may alter the expression of many of these genes in ways that can result in differential presentations of psychosis in the human PWS population. As we have determined here, the hippocampus and ACC are specifically dysregulated in this animal model, and future studies can examine how this mutation affects the transcriptome in those regions, with a particular focus on the genes implicated in Table S2. Such studies could provide clues as to the mechanism by which *Snord116* deletion can induce a psychosis-like state, and perhaps evidence alterations in genes and gene modules specific to PWS, as opposed to other models of psychotic illness based on pharmacological means or individual schizophrenia/bipolar-associated genes. 

Taken together, the results demonstrated here show that *Snord116* deletion can recapitulate several of the behavioral phenotypes associated with animal model or human presentations of psychosis. As the *Snord116*^p−/m+^ mice have only a microdeletion of a single paternally inherited gene within the region, this raises interesting questions about the unique role of *Snord116* in the expression of psychosis and could potentially be used as a distinct target for both PWS and psychosis treatments. Future studies with which to assess the effects of the deletion of or alterations in *Snord116* downstream targets, or the externally manipulated upregulation of *Snord116*, could provide clues to the precise mechanisms underlying the emergence of specific behavioral features associated with PWS and psychosis. 

## Data Availability

The raw data supporting the conclusions of this article will be made available by the authors on request.

## Supplementary Materials

The following supporting information can be downloaded at: https://www.mdpi.com/article/10.3390/genes16080863/s1, Table S1: Descriptive Statistics; Table S2: Gene Targets of Snord116 Associated with Psychosis [48,49,50,51,52,53,54,55,56,57,58,59,60,61,62,63,64,65,66,67,68,69,70,71].

## Abbreviations

The following abbreviations are used in this manuscript:

PWS	Prader–Willi syndrome
ACC	Anterior cingulate cortex
PPI	Prepulse inhibition
WT	Wild type
