# Supplementary material for: Altered Behavior and Neuronal Activity with Paternal Snord116 Deletion
Source: Genes (Basel). 2025 Jul 24;16(8):863. doi: 10.3390/genes16080863 (PMC12385964; doi:10.3390/genes16080863)
Supplement: Supplementary file 1 [file genes-16-00863-s001.zip › genes-3733221-supplementary.pdf]

**Table S1**

**Descriptive Statistics**

| Task | Units | WT | | | *Snord116*$^{p-/m+}$ | | |
|---|---|---|---|---|---|---|---|
| | | N | Mean | SD | N | Mean | SD |
| cFos analysis - ACC (Male) | % of control | 17 | 96.92 | 23.78 | 12 | 136.68 | 34.01 |
| cFos analysis - ACC (Female) | % of control | 19 | 94.70 | 30.63 | 9 | 134.87 | 39.97 |
| cFos analysis - DG (Male) | % of control | 19 | 101.71 | 22.45 | 12 | 108.84 | 26.62 |
| cFos analysis - DG (Female) | % of control | 19 | 98.29 | 40.71 | 10 | 127.72 | 33.65 |
| cFos analysis - CA3 (Male) | % of control | 19 | 100.35 | 30.94 | 12 | 101.59 | 48.71 |
| cFos analysis - CA3 (Female) | % of control | 19 | 99.65 | 33.15 | 10 | 107.31 | 16.89 |
| cFos analysis - CA1 (Male) | % of control | 19 | 91.34 | 36.19 | 11 | 93.50 | 48.16 |
| cFos analysis - CA1 (Female) | % of control | 19 | 108.66 | 37.26 | 10 | 138.27 | 58.39 |
| Open field - total activity (Male) | cm | 19 | 3743.15 | 958.91 | 12 | 3456.65 | 637.61 |
| Open field - total activity (Female) | cm | 19 | 4462.85 | 872.03 | 10 | 3582.58 | 770.90 |
| Open field - center time (Male) | seconds | 19 | 24.08 | 16.47 | 12 | 21.96 | 12.14 |
| Open field - center time (Female) | seconds | 18 | 20.80 | 8.39 | 10 | 19.07 | 8.63 |
| Open field - thigmotaxis (Male) | seconds | 19 | 337.93 | 63.29 | 12 | 378.38 | 60.74 |
| Open field - thigmotaxis (Female) | seconds | 19 | 372.70 | 68.31 | 10 | 359.24 | 83.30 |
| Social Memory (Male) | time difference (seconds) | 19 | 8.47 | 18.13 | 12 | 22.25 | 17.38 |
| Social Memory (Female) | time difference (seconds) | 19 | 13.05 | 14.51 | 10 | 24.20 | 10.24 |
| Spontaneous Alternation (Male) | % alternation | 19 | 76.70 | 15.62 | 12 | 70.76 | 14.67 |
| Spontaneous Alternation (Female) | % alternation | 19 | 70.08 | 12.41 | 10 | 67.89 | 17.67 |
| Prepulse Inhibition - 72db (Male) | % inhibition | 19 | 19.72 | 26.14 | 12 | 22.65 | 16.36 |
| Prepulse Inhibition - 72db (Female) | % inhibition | 19 | 10.71 | 29.86 | 9 | 0.58 | 29.56 |

| Task | Units | WT | | | *Snord116*$^{p-/m+}$ | | |
|---|---|---|---|---|---|---|---|
| | | N | Mean | SD | N | Mean | SD |
| Prepulse Inhibition - 74db (Male) | % inhibition | 19 | 38.26 | 22.85 | 12 | 47.64 | 24.81 |
| Prepulse Inhibition - 74db (Female) | % inhibition | 19 | 37.22 | 21.33 | 10 | 0.37 | 49.67 |
| Prepulse Inhibition - 78db (Male) | % inhibition | 19 | 65.05 | 22.69 | 12 | 68.91 | 10.67 |
| Prepulse Inhibition - 78db (Female) | % inhibition | 19 | 59.05 | 20.59 | 10 | 44.15 | 24.15 |
| Prepulse Inhibition - 82db (Male) | % inhibition | 19 | 73.94 | 15.37 | 11 | 75.26 | 3.86 |
| Prepulse Inhibition - 82db (Female) | % inhibition | 19 | 65.51 | 17.02 | 10 | 56.63 | 21.59 |
| Contextual Fear Conditioning (Male) | % inhibition | 19 | 41.86 | 17.05 | 12 | 54.48 | 23.87 |
| Contextual Fear Conditioning (Female) | % freezing | 18 | 29.93 | 13.58 | 10 | 57.99 | 18.04 |
| Cued Fear Conditioning - Pre-CS (Male) | % freezing | 15 | 7.59 | 3.33 | 12 | 17.60 | 9.87 |
| Cued Fear Conditioning - Pre-CS (Female) | % freezing | 18 | 5.82 | 3.80 | 10 | 19.64 | 10.38 |
| Cued Fear Conditioning - CS (Male) | % freezing | 19 | 64.96 | 16.80 | 12 | 65.49 | 13.02 |
| Cued Fear Conditioning - CS (Female) | % freezing | 19 | 60.06 | 21.76 | 10 | 66.88 | 16.36 |

**Table S2**

**Gene Targets of Snord116 Associated with Psychosis**

| Gene | Protein Name | Human Findings | Animal Models | References |
|---|---|---|---|---|
| *CDK5R1* | p35 | Decreased expression in hippocampus and PFC in SZ | Impaired PPI and spatial memory formation in female +/- mice; impaired spatial working memory and social interaction in male +/- mice | Engmann, et al., 2011[1] |
| *DUSP4* | MKP2 | Decreased expression in cerebellar vermis in SZ | | Kyosseva, et al., 1999[2] |
| *GRM7* | mGluR7 | rs1516569, rs3749380, rs12491620, and rs1450099 variants associated with SZ; other variants associated with antipsychotic response | KO mouse shows impaired social memory, fear conditioning | Ohtsuki, et al., 2008[3]; Shibata, et al., 2009[4]; Liang, et al., 2020[5]; Fisher, et al., 2021[6] |
| *HSPA1L* | HSP70 | rs2227956 and rs2075799 variants associated with SZ | | Kim, et al., 2008[7] |
| *IL18R1* | Interleukin 18 receptor 1 | rs1035130 variant associated with SZ; decreased serum expression in SZ | | Xu, et al., 2016[8] |
| *IL1RAPL1* | Interleukin 1 receptor accessory protein like 1 | Missense variant associated with early-onset SZ | | Kanwal, et al., 2024[9] |
| *KCTD10* | Potassium channel tetramerization domain containing 10 | p.C124W de novo mutation associated with SZ | Het mouse shows impaired social interaction, social memory, spatial memory in MWM and Y-maze, increased anxiety in open field and light-dark transition test. Mice with C124W mutation show decreased dendritic spines on secondary basal dendrites on cortical and secondary apical dendrites on | Mu, et al., 2024[10] |

| Gene | Protein Name | Human Findings | Animal Models | References |
|---|---|---|---|---|
| | | | hippocampal CA1 pyramidal cells | |
| *KIF24* | Kinesin family member 24 | Missense mutation found in sporadic SZ case | | Giupponi, et al., 2014[11] |
| *LUZP2* | Leucine zipper protein 2 | Closest gene to SZ GWAS-associated polymorphism | | Schizophrenia Working Group of the Psychiatric Genomics Consortium, 2014[12] |
| *NEB* | Neubulin | snp in promoter region of LASP1, a splice variant of NEB, associated with schizophrenia in a Korean population | LASP1, a splice variant of NEB, is downregulated in the hippocampus after MK-801 administration in mice | Joo, et al., 2013[13] |
| *PAX5* | Pax-5 | Decreased copy number intensity in hippocampal CA2/3 in SZ | | Sheng, et al., 2012[14] |
| *PAX6* | Pax-6 | Inherited mutation associated with psychosis in a single family. Potential association of the high-activity PAX6 variant with paranoid schizophrenia | Forebrain-specific KO mouse shows impaired short- and long-term memory recall, sensorimotor integration | Stober, et al., 1999[15]; Heyman, et al., 1999[16]; Tuoc, et al., 2009[17] |
| *PIDD1* | p53-induced death domain protein 1 | Rare variants associated with psychosis | | Zaki, et al., 2021[18] |
| *PLAGL1* | PLAG1 like zinc finger 1 | Rare epivariations associated with ASD | | Garg and Sharp, 2019[19] |
| *PPP1R17* | protein phosphatase 1 regulatory subunit 17 | Decreased transcription in cortical neurons derived from SZ fibroblasts | | Tiihonen, et al., 2019[20] |
| *PTN* | Pleiotrophin | Variants associated with SZ | | Schizophrenia Working Group of the Psychiatric Genomics Consortium, 2014[12] |
| *PTX3* | Pentraxin 3 | Decreased serum expression in SZ | | Weber, et al., 2015[21] |

| Gene | Protein Name | Human Findings | Animal Models | References |
|---|---|---|---|---|
| *RAB7A* | Rab-7a | Interacts with dysregulated proteins in several pathways in SZ DLPFC | | Vera-Montecinos, et al., 2023[22] |
| *RIMKLA* | Ribosomal Modification Protein RimK Like Family Member A | | KO mice show impaired short-term novel object recognition, reduced brain NAAG | Becker, et al., 2021[23] |
| *ZIC2* | Zic2 | Ins239H, A95T, R409P and S444R mutations associated with SZ | Hypomorphic mice show impaired cued/contextual fear conditioning, spatial working memory, PPI, increased locomotion | Hatayama, et al., 2011[24] |

1. Engmann, O., Hortobagyi, T., Pidsley, R., Troakes, C., Bernstein, H.G., Kreutz, M.R., Mill, J., Nikolic, M., and Giese, K.P. (2011). Schizophrenia is associated with dysregulation of a Cdk5 activator that regulates synaptic protein expression and cognition. Brain *134*, 2408-2421. 10.1093/brain/awr155.
2. Kyosseva, S.V., Elbein, A.D., Griffin, W.S., Mrak, R.E., Lyon, M., and Karson, C.N. (1999). Mitogen-activated protein kinases in schizophrenia. Biol Psychiatry *46*, 689-696. 10.1016/s0006-3223(99)00104-3.
3. Ohtsuki, T., Koga, M., Ishiguro, H., Horiuchi, Y., Arai, M., Niizato, K., Itokawa, M., Inada, T., Iwata, N., Iritani, S., et al. (2008). A polymorphism of the metabotropic glutamate receptor mGluR7 (GRM7) gene is associated with schizophrenia. Schizophr Res *101*, 9-16. 10.1016/j.schres.2008.01.027.
4. Shibata, H., Tani, A., Chikuhara, T., Kikuta, R., Sakai, M., Ninomiya, H., Tashiro, N., Iwata, N., Ozaki, N., and Fukumaki, Y. (2009). Association study of polymorphisms in the group III metabotropic glutamate receptor genes, GRM4 and GRM7, with schizophrenia. Psychiatry Res *167*, 88-96. 10.1016/j.psychres.2007.12.002.
5. Liang, W., Yu, H., Su, Y., Lu, T., Yan, H., Yue, W., and Zhang, D. (2020). Variants of GRM7 as risk factor and response to antipsychotic therapy in schizophrenia. Transl Psychiatry *10*, 83. 10.1038/s41398-020-0763-4.
6. Fisher, N.M., AlHashim, A., Buch, A.B., Badivuku, H., Samman, M.M., Weiss, K.M., Cestero, G.I., Does, M.D., Rook, J.M., Lindsley, C.W., et al. (2021). A GRM7 mutation associated with developmental delay reduces mGlu7 expression and produces neurological phenotypes. JCI Insight *6*. 10.1172/jci.insight.143324.
7. Kim, J.J., Mandelli, L., Lim, S., Lim, H.K., Kwon, O.J., Pae, C.U., Serretti, A., Nimgaonkar, V.L., Paik, I.H., and Jun, T.Y. (2008). Association analysis of heat shock protein 70 gene polymorphisms in schizophrenia. Eur Arch Psychiatry Clin Neurosci *258*, 239-244. 10.1007/s00406-007-0791-6.
8. Xu, Y., Yue, W., Shugart, Y.Y., Yuan, J., Wang, G., Wang, H.Z., Lehrman, B., Zhang, F., and Zhang, D. (2016). Potential involvement of the interleukin-18 pathway in schizophrenia. J Psychiatr Res *74*, 10-16. 10.1016/j.jpsychires.2015.12.013.
9. Kanwal, A., Pardo, J.V., and Naz, S. (2022). RGS3 and IL1RAPL1 missense variants implicate defective neurotransmission in early-onset inherited schizophrenias. J Psychiatry Neurosci *47*, E379-E390. 10.1503/jpn.220070.
10. Mu, C., Liu, P., Liu, L., Wang, Y., Liu, K., Li, X., Li, G., Cheng, J., Bu, M., Chen, H., et al. (2024). KCTD10 p.C124W variant contributes to schizophrenia by attenuating LLPS-mediated synapse formation. Proc Natl Acad Sci U S A *121*, e2400464121. 10.1073/pnas.2400464121.
11. Guipponi, M., Santoni, F.A., Setola, V., Gehrig, C., Rotharmel, M., Cuenca, M., Guillin, O., Dikeos, D., Georgantopoulos, G., Papadimitriou, G., et al. (2014). Exome sequencing in 53 sporadic cases of schizophrenia identifies 18 putative candidate genes. PLoS One *9*, e112745. 10.1371/journal.pone.0112745.
12. Schizophrenia Working Group of the Psychiatric Genomics, C. (2014). Biological insights from 108 schizophrenia-associated genetic loci. Nature *511*, 421-427. 10.1038/nature13595.
13. Joo, J., Lee, S., Nah, S.S., Kim, Y.O., Kim, D.S., Shim, S.H., Hwangbo, Y., Kim, H.K., Kwon, J.T., Kim, J.W., et al. (2013). Lasp1 is down-regulated in NMDA receptor antagonist-treated mice and implicated in human schizophrenia susceptibility. J Psychiatr Res *47*, 105-112. 10.1016/j.jpsychires.2012.09.005.
14. Sheng, G., Demers, M., Subburaju, S., and Benes, F.M. (2012). Differences in the circuitry-based association of copy numbers and gene expression between the hippocampi of patients with

schizophrenia and the hippocampi of patients with bipolar disorder. Arch Gen Psychiatry *69*, 550-561. 10.1001/archgenpsychiatry.2011.1882.
15. Stober, G., Syagailo, Y.V., Okladnova, O., Jungkunz, G., Knapp, M., Beckmann, H., and Lesch, K.P. (1999). Functional PAX-6 gene-linked polymorphic region: potential association with paranoid schizophrenia. Biol Psychiatry *45*, 1585-1591. 10.1016/s0006-3223(99)00024-4.
16. Heyman, I., Frampton, I., van Heyningen, V., Hanson, I., Teague, P., Taylor, A., and Simonoff, E. (1999). Psychiatric disorder and cognitive function in a family with an inherited novel mutation of the developmental control gene PAX6. Psychiatr Genet *9*, 85-90. 10.1097/00041444-199906000-00006.
17. Tuoc, T.C., Radyushkin, K., Tonchev, A.B., Pinon, M.C., Ashery-Padan, R., Molnar, Z., Davidoff, M.S., and Stoykova, A. (2009). Selective cortical layering abnormalities and behavioral deficits in cortex-specific Pax6 knock-out mice. J Neurosci *29*, 8335-8349. 10.1523/JNEUROSCI.5669-08.2009.
18. Zaki, M.S., Accogli, A., Mirzaa, G., Rahman, F., Mohammed, H., Porras-Hurtado, G.L., Efthymiou, S., Maqbool, S., Shukla, A., Vincent, J.B., et al. (2021). Pathogenic variants in PIDD1 lead to an autosomal recessive neurodevelopmental disorder with pachygyria and psychiatric features. Eur J Hum Genet *29*, 1226-1234. 10.1038/s41431-021-00910-0.
19. Garg, P., and Sharp, A.J. (2019). Screening for rare epigenetic variations in autism and schizophrenia. Hum Mutat *40*, 952-961. 10.1002/humu.23740.
20. Tiihonen, J., Koskuvi, M., Storvik, M., Hyotylainen, I., Gao, Y., Puttonen, K.A., Giniatullina, R., Poguzhelskaya, E., Ojansuu, I., Vaurio, O., et al. (2019). Sex-specific transcriptional and proteomic signatures in schizophrenia. Nat Commun *10*, 3933. 10.1038/s41467-019-11797-3.
21. Weber, N.S., Larsen, R.A., Yolken, R.H., Cowan, D.N., Boivin, M.R., and Niebuhr, D.W. (2015). Predictors of the Onset of Schizophrenia in US Military Personnel. J Nerv Ment Dis *203*, 319-324. 10.1097/NMD.0000000000000285.
22. Vera-Montecinos, A., Rodriguez-Mias, R., Vila, E., Villen, J., and Ramos, B. (2023). Analysis of networks in the dorsolateral prefrontal cortex in chronic schizophrenia: Relevance of altered immune response. Front Pharmacol *14*, 1003557. 10.3389/fphar.2023.1003557.
23. Becker, I., Wang-Eckhardt, L., Lodder-Gadaczek, J., Wang, Y., Grunewald, A., and Eckhardt, M. (2021). Mice deficient in the NAAG synthetase II gene Rimkla are impaired in a novel object recognition task. J Neurochem *157*, 2008-2023. 10.1111/jnc.15333.
24. Hatayama, M., Ishiguro, A., Iwayama, Y., Takashima, N., Sakoori, K., Toyota, T., Nozaki, Y., Odaka, Y.S., Yamada, K., Yoshikawa, T., and Aruga, J. (2011). Zic2 hypomorphic mutant mice as a schizophrenia model and ZIC2 mutations identified in schizophrenia patients. Sci Rep *1*, 16. 10.1038/srep00016.
